# Predictive Value of PRISM-4, PIM-3, CRP, Albumin, CRP/Albumin Ratio and Lactate in Critically Ill Children

**DOI:** 10.3390/children10111731

**Published:** 2023-10-26

**Authors:** Gazi Arslan, Tolga Besci, Göktuğ Özdemir, Gültaç Evren, Hande Ilgaz Tüzen, Pınar Prencuva, Bengisu Turan, Ebru Melek Benligül

**Affiliations:** Department of Pediatrics, Division of Pediatric Intensive Care, Faculty of Medicine, Dokuz Eylül University, Konak 35220, Turkey; tolga.besci@deu.edu.tr (T.B.); goktug.ozdemir@deu.edu.tr (G.Ö.); gultac.evren@deu.edu.tr (G.E.); hande.ilgaz@deu.edu.tr (H.I.T.); pinar.prencuva@deu.edu.tr (P.P.); bengisu.turan@deu.edu.tr (B.T.); melek.benligul@deu.edu.tr (E.M.B.)

**Keywords:** mortality, pediatric critical care, albumin, lactate, sepsis

## Abstract

The accurate prediction of the prognosis for critically ill children is crucial, with the Pediatric Index of Mortality (PIM) and Pediatric Risk of Mortality (PRISM) being extensively utilized for this purpose. Albumin, C-reactive protein (CRP), and lactate levels, which are indicative of inflammation and circulatory status in critically ill children, have not been incorporated into existing scoring systems. This retrospective cohort study evaluated the association between biological markers and the clinical outcomes in children with critical illnesses. PRISM-4 and PIM-3 death probability (DP), albumin, lactate, CRP, and CRP/albumin ratio were recorded upon admission. The accuracy of the indexes in predicting mortality were assessed by calculating the area under the curve (AUC). There were 942 patients included and the 28-day mortality rate was 7.9%. The AUC for PRISM-4, PIM-3, CRP, CRP/albumin ratio, albumin, and lactate were 0.923, 0.896, 0.798, 0.795, 0.751, 0.728, respectively. The findings in the subgroup analysis of septic patients were similar to those found in the overall population. Although CRP, CRP/albumin ratio, albumin, and lactate levels are all linked to mortality in children, CRP and the CRP/albumin ratio have lower predictive values than albumin and lactate. Incorporation of albumin and lactate into scoring systems will improve predictability.

## 1. Introduction

The accurate prediction of the prognosis of pediatric patients receiving treatment in pediatric intensive care units (PICUs) holds significant importance as it plays a vital role in guiding the therapy. Mortality prediction models have emerged as a crucial instrument for assessing the quality of intensive care [1]. Several factors unrelated to standard of care, including the patient’s diagnosis, underlying health condition, and severity of current illness, contribute to the probability of mortality for an individual patient. In addition, PICUs provide medical care for children with diverse organ failures, as well as surgical patients requiring treatment for trauma or post-operative monitoring [2]. The patient population under consideration has a significant degree of heterogeneity, frequently presenting with inflammatory or circulatory disorders. Numerous scoring methods have been devised for the assessment of children, although the Pediatric Index of Mortality (PIM) and Pediatric Risk of Mortality (PRISM) scoring systems have emerged as the prevailing methodologies employed in contemporary practice. PRISM is a prognostic tool that originated in PICUs within the United States in 1988. Its most recent update occurred in 2016, and it integrates physiological parameters alongside biochemical and hematological indicators. The PIM method, conversely, was developed utilizing data collected from PICUs in the United Kingdom, Ireland, New Zealand, and Australia. This method, last updated in 2015, aims to ascertain the projected mortality rate by incorporating the underlying diagnosis, physiological variables, and select laboratory data, such as base deficit. The PIM 3 update included two extensive international outcome registries and enhanced the ability to predict mortality, particularly in patients identified as low-risk [3,4].

Although there is considerable diversity in the primary diagnoses of patients admitted to the PICU, a majority of these patients present with concurrent cardiovascular, respiratory, or inflammatory conditions. Several indicators of inflammation have been identified, including acute phase proteins such as C-reactive protein (CRP), albumin, and amyloid A. Additionally, blood infection markers such as procalcitonin have been described [5,6]. Among the various markers, CRP and albumin highly reflects inflammation in critically ill patients [7,8]. Lactate plays a significant role in cellular energy metabolism, and its concentration in circulation tends to rise in critically ill pediatric patients having hypoperfusion, hyperinflammation, hypoxia, sepsis, shock, and multiple organ failure [9]. Furthermore, it can be concluded that there exists a correlation between the levels of CRP, albumin, and lactate and the degree of inflammation, thereby suggesting their potential utility as prognostic indicators. Despite the existence of research indicating the association between mortality and markers such as CRP, albumin, CRP/albumin ratio, and lactate, it is noteworthy that current scoring systems have not yet incorporated these markers [10,11,12,13]. Furthermore, the tests for CRP, albumin, and blood lactate levels are highly accessible and offer significant insights into the inflammatory response, nutritional status, and circulatory functions of patients in critical states [14,15,16].

While prognostic markers may not possess the same level of predictive capability as scoring systems in determining mortality, they do serve a valuable purpose in diagnosing and monitoring specific diseases within a population. Additionally, they can provide guidance for health strategies and the allocation of resources. Furthermore, prognostic markers have the potential to not only facilitate the diagnosis of inflammatory conditions such as sepsis, but also predict the likelihood of survival in septic patients. Timely and accurate identification of these individuals enables the implementation of an appropriate intervention upon being admitted to the PICU, potentially leading to enhanced outcomes.

The purpose of this research was to investigate the potential associations between different biological indicators, including CRP, lactate, albumin, and CRP/albumin ratio and the clinical outcomes of children with critical illness who were admitted to the PICU.

## 2. Materials and Methods

### 2.1. Study Design and Patients

The present research is a retrospective cohort study performed within a tertiary PICU. The acquisition of written informed consent was not pursued due to the retrospective nature of the study. All individuals between the ages of one month and 18 years who have been admitted to a general (medical, cardiac and surgery) PICU from 1 January 2018 to 31 December 2022 were screened for inclusion, and children with mortality scores and laboratory findings were included in the study. The serum albumin levels are also influenced by liver failure and malnutrition. These patients exhibit decreased levels of albumin independent of their inflammatory state, and the administration of blood products may potentially increase their blood albumin levels. Therefore, children who exhibited liver failure, severe malnutrition, and had received blood products prior to admission were excluded from the study. Only the initial admittance of the data input was documented within the established research period.

### 2.2. Data Collection and Statistical Analysis

The study collected data on the primary and concomitant diagnoses of patients upon their arrival at the PICU, using the diagnostic codes from the Australian and New Zealand Pediatric Intensive Care registry [17]. Additionally, information regarding underlying diseases, clinical and demographic variables were also collected. The laboratory results of CRP (measured using an immunoluminometric test, with a reference range of 0–8 mg/L), albumin (measured using a colorimetric assay, with a reference range of 3.4–5.4 g/dL), and venous blood lactate (measured in mmol/L) were documented within the initial 24-h period following admission. Additionally, prognostic indicators including the PRISM-4 death probability (PRISM-4 DP) and PIM-3 death probability (PIM-3 DP) were also recorded. The collection of all variables occurred within the initial 24-h period following admission. All patients were evaluated according to the Surviving Sepsis Guidelines for the diagnosis of primary or accompanying sepsis upon admission [18].

The data analysis was conducted utilizing SPSS 22.0 software (SPSS, Chicago, IL, USA). The researchers conducted statistical analyses to compare the demographic and clinical characteristics of the patients. The Mann–Whitney test was utilized to analyze quantitative variables, whereas the Pearson’s chi-squared test was employed to examine qualitative variables. The receiver operating characteristic (ROC) curve was used to analyze the prediction power of prognostic indicators. The assessment of the overall accuracy of the index was conducted by utilizing the area under the receiver operating characteristic curve (AUC) and its corresponding confidence interval (CI). The Youden index was employed to ascertain the optimal cut-off values. The 28-day mortality of the two patient groups was evaluated and analyzed using a Kaplan–Meier curve, using preset cut-off values. The nonparametric log-rank test was used to evaluate and compare the survival curves of the two groups. *p*-values of less than 0.05 is considered statistically significant.

## 3. Results

### 3.1. Study Population of All Patients

**Figure 1:**
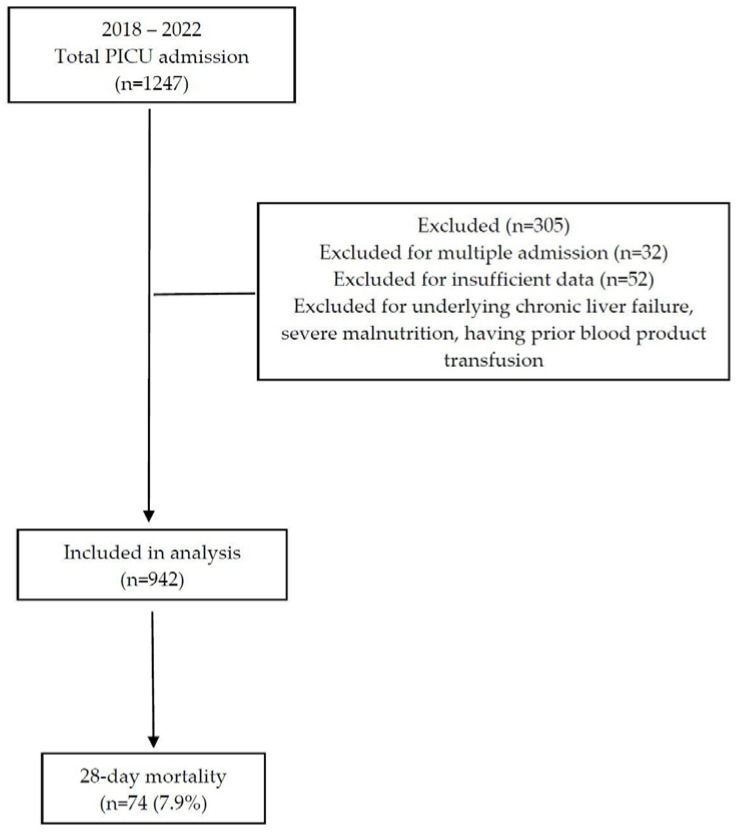
A flowchart depicting the process of patient selection.

Throughout the designated study period, an overall count of 1247 individuals was admitted to the PICU. A subset of 32 patients from the overall sample was excluded from the analysis due to insufficient data. Additionally, 52 patients were excluded as a result of duplicate admissions. Furthermore, 221 patients with chronic liver failure, severe malnutrition, and prior blood product administration were excluded. As a result, the ultimate examination encompassed a cumulative number of 942 individuals (Figure 1).

Demographic variables and clinical characteristics of patients are summarized in Table 1. The 28-day mortality rate in the PICU was found to be 7.9%, while the overall in-hospital mortality rate was 11.1%. The study revealed that mortality rates were higher in patients who exhibited primary or accompanying sepsis (*p* < 0.01), utilized invasive mechanical ventilation (*p* < 0.01), received vasoactive medications (*p* < 0.01), and had underlying cancer (*p* < 0.01). Among the patients included, the primary diagnoses for children admitted to the PICU were respiratory failure (29.8%), surgical (21.3%), neurologic (13.3%), trauma (11.3%), cardiovascular (9.5%), septic shock (6.7%), and other diagnoses (8.1%).

**Figure 2:**
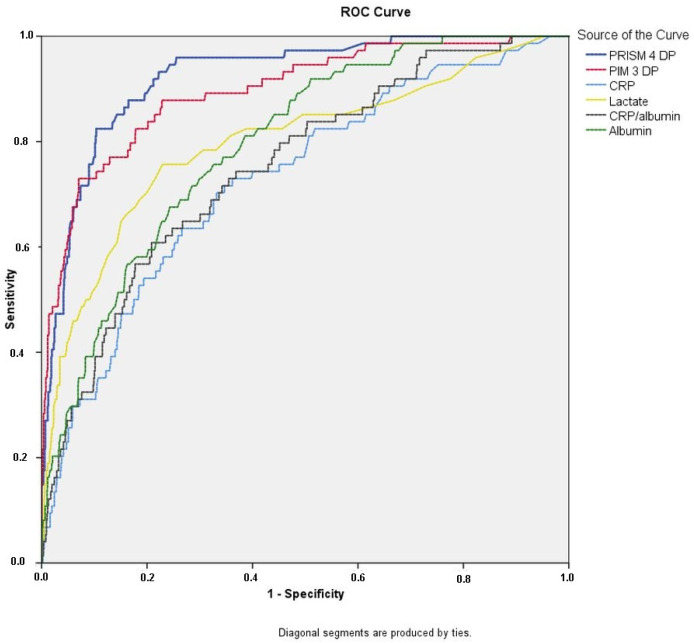
Comparison of ROC curves of PRISM-4 DP, PIM-3 DP, CRP, CRP/albumin ratio, albumin and blood lactate levels of critically ill pediatric patients. ROC = receiver operating characteristics, PRISM-4 DP = pediatric risk of mortality death probability, PIM-3 DP = pediatric index of mortality death probability, CRP = C-reactive protein.

The analysis was conducted on a sample population including 942 patients. The AUC, and *p*-values were calculated for prognostic variables including mortality predicting scores, CRP, CRP/albumin ratio, albumin, and lactate levels. This analysis was performed separately for survivors and non-survivors. These results are presented in Figure 2 and Table 2. The AUC values for CRP, CRP/albumin ratio, albumin, and lactate were 0.712, 0.736, 0.784, and 0.801, respectively. The established thresholds for the CRP/albumin ratio, albumin concentration, and blood lactate levels were determined to be 19.64, 2.9 g/dL, and 3.2 mmol/L, respectively.

### 3.2. Study Population of Septic and Non-septic Patients

All patients in the study group also evaluated for principal or concomitant sepsis according to Surviving Sepsis Guidelines and the patients were designated as septic (*n* = 173; 37% females, age 52 [9.5–127] month), and non-septic (*n* = 769; 41.6% females, age 28 [9–96] month). Out of the total population of septic patients, 64 of them (37%) presented with a primary sepsis diagnosis, whereas 109 of them (63%) had a concomitant sepsis diagnosis upon admission. Table 3 shows the comparative analysis of data related to demographic characteristics, prognostic indices, and biological markers, along with their appropriate *p* values, for patients diagnosed with sepsis and those without sepsis.

**Figure 3:**
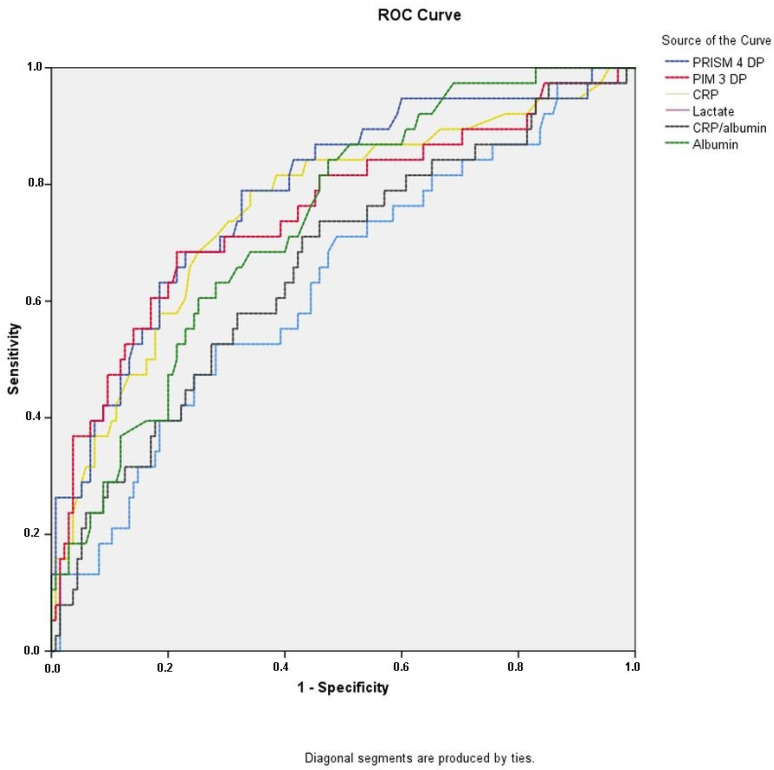
Comparison of ROC curves of PRISM-4 DP, PIM-3 DP, CRP, CRP/albumin ratio, albumin and lactate of pediatric patients who were septic. ROC = receiver operating characteristics, PRISM-4 DP = pediatric risk of mortality death probability, PIM-3 DP = pediatric index of mortality death probability, CRP = C-reactive protein.

Within the group of patients, consisting of 173 individuals, who had sepsis during admission, several biomarkers showed significant predictive efficacy in determining the survival outcome of sepsis. Table 4 demonstrates that albumin (*p* < 0.01), CRP/albumin ratio (*p* < 0.01), lactate (*p* < 0.01), and to a lesser degree CRP (*p* = 0.015) were identified as potential indicators of survival in sepsis. The AUC values for biomarkers were shown in Figure 3 and Table 5.

## 4. Discussion

The findings of this study demonstrate a statistically significant association between higher lactate, CRP, CRP/albumin ratio, reduced albumin levels upon admission to the PICU, and an increased probability of mortality. The highest AUC values were observed for blood lactate level, albumin, CRP/albumin ratio, and CRP, in that order. The predictive performance of the CRP/albumin ratio in terms of mortality was shown to be superior to that of CRP alone. However, it was observed that both lactate and albumin demonstrated better predictive capabilities in relation to mortality when compared to both the CRP/albumin ratio and CRP.

Due to the incorporation of multiple physiological and biological parameters within their equations, mortality indices are anticipated to exhibit superior predictive capabilities compared to single variables. Nevertheless, it is important to note that certain physiological variables and biomarkers, including albumin, CRP, and lactate, have the potential to be significant factors on their own in the prediction of mortality among children in a critical condition. Moreover, it is important to note that these biological markers possess the capacity to predict the probability of survival in individuals with hyperinflammatory conditions, such as sepsis. The rapid detection highlights the importance of promptly and accurately identifying these individuals, as it allows for the implementation of appropriate treatments upon admission to the PICU. This, in turn, has the potential to result in improved outcomes. In addition, by incorporating these variables into the equations of mortality indices, their predictive capacity may be enhanced.

The association between high CRP levels and a range of conditions, including severe sepsis and inflammatory diseases, has been demonstrated in critically ill patients [19]. Furthermore, CRP serves as a prognostic indicator within the critical care environment [20]. However, some studies showed that CRP alone did not successfully predict mortality in septic patients [21]. Conversely, hypoalbuminemia is frequently observed in critically ill individuals, and there exists a correlation between serum albumin levels and heightened mortality rates among acutely ill pediatric populations [22]. In a prospective study conducted in a PICU with a sample size of 271 participants, it was found that albumin levels below 3.0 gr/dL were significantly associated with increased mortality rates, prolonged mechanical ventilation, and reduced likelihood of discharge from the PICU [11]. The findings of this study demonstrate a noteworthy association between reduced levels of albumin and mortality rates in critically ill and septic children. This association is supported by the AUC values of 0.795 and 0.734, respectively. Numerous studies have demonstrated a significant correlation between mortality rates and the concurrent utilization of CRP and albumin, specifically in the context of critically ill patients [23,24]. Nevertheless, the comparative analysis of the prognostic efficacy between CRP/albumin and albumin was not conducted in these studies. There is only one research study carried out on adults that has demonstrated the prognostic efficacy of the CRP/albumin ratio, with albumin possessing an essential role in this regard [5]. In a retrospective study conducted on a pediatric population consisting of 178 patients with a high mortality rate (20.8%) in the PICU, it was observed that the predictive value of the ratio between CRP and albumin was superior to that of CRP and albumin as individual markers [15]. In a prospective pediatric study (*n* = 100) investigating the prognostic value (death or organ failure) of albumin and CRP, the ROC curves for albumin and CRP as a predictor had AUC values of 0.92 and 0.59, respectively, but the combination of albumin and CRP showed better sensitivity (96.83%) and specificity (91.89%) in determining the unfavorable prognosis [25]. The present study provides evidence that the prognostic effectiveness of albumin surpasses that of the CRP/albumin ratio in critically ill pediatric individuals. While the CRP/albumin ratio exhibits some predictive value for mortality in critically ill children, its relative strength is diminished in comparison to albumin. Therefore, compared to the majority of studies conducted on adult populations, depending exclusively on the CRP/albumin ratio as a sole indicator of mortality in critically ill pediatric patients is not recommended.

The integration of the CRP/albumin ratio provides an evaluation that incorporates the data obtained from CRP and albumin, leading to an indicator that exhibits a positive correlation with infection and has been employed as a prognostic instrument for assessing outcomes in patients diagnosed with sepsis. More specifically, a higher ratio indicates increased levels of inflammation [24]. In a study conducted among the adult population, researchers observed that septic patients who underwent early goal-directed therapy exhibited a higher predictive value for mortality when considering the CRP/albumin ratio as opposed to CRP alone (AUC for CRP/albumin ratio = 0.6211, AUC for CRP alone = 0.5620) [23]. In a neonatal study, researchers discovered that the CRP/albumin ratio serves as an independent indicator for determining the likelihood and intensity of sepsis. The AUC values for sepsis and severe sepsis were determined to be 0.74 and 0.70, respectively [26]. In the present study, an examination of subgroup analyses was conducted in patients diagnosed with sepsis. Consistent with findings observed in the general PICU population, the prognostic significance of lactate and albumin levels in predicting mortality among septic patients surpasses that of both the CRP/albumin ratio and CRP. Furthermore, the AUC of CRP/albumin and CRP in septic patients exhibited a lower value compared to the AUC of patients within the overall population.

Elevated blood lactate levels upon admission or impaired lactate clearance have been found to be correlated with unfavorable outcomes in both septic and other critically ill pediatric patients [9,27,28,29]. Consistent with our findings, a study conducted in critically ill children (*n* = 1109) demonstrated that blood lactate level exhibited an AUC of 0.79 and showed a sensitivity of 61% and a specificity of 86% in predicting mortality when the optimal cut-off value of 5.55 mmol/L was utilized [28]. In a different study that assessed children who were thought to have septic shock (*n* = 1299) in a pediatric emergency department, a blood lactate level higher than 4 mmol/L was strongly linked to mortality within 30 days [29]. In a limited-scale prospective observational study involving a sample size of 30 individuals, it was found that an elevated blood lactate level exceeding 5 mmol/L served as a reliable indicator for predicting mortality among children who were admitted to the PICU and diagnosed with septic shock [30]. In accordance with our investigation, it has been observed that the occurrence of heightened lactate levels in children admitted to the PICU serves as a noteworthy predictor of mortality. This holds true for cases of sepsis as well as for all the critically ill children.

This research has several limitations. Despite an appropriate amount of cases, the single-center nature of this study limits the generalizability of the findings to other populations. Additionally, the study is also retrospective and observational in nature. Furthermore, while subgroup analysis was conducted specifically on septic children, our evaluation encompassed a diverse group of patients from the PICU.

This study investigated the predictive value of albumin, lactate, CRP, and CRP/albumin levels in a large sample of pediatric patients. Despite the fact that the predictive value of the CRP/albumin ratio is greater than that of CRP alone, it is not as significant as lactate and albumin. While albumin, lactate, and the CRP/albumin ratio may not possess robust predictive capabilities as mortality indicators that encompass a wide range of physiological and laboratory data, they can still be utilized to identify high-risk patients at an earlier stage, facilitate the administration of suitable treatment, and assess the response to treatment. To validate these findings in specific pediatric patient populations, further prospective multicenter studies are required.

## 5. Conclusions

Consistent with prior researches, it has been observed that levels of CRP, CRP/albumin ratio, albumin, and blood lactate levels are individually linked to mortality rates in all children admitted to the PICU and specifically in septic children. Nevertheless, it should be noted that both CRP and the CRP/albumin ratio exhibit lower predictive values compared to albumin and blood lactate levels. Given the relatively diminished predictive capacity of the CRP/albumin ratio compared to albumin alone, it could be stated that utilizing this ratio may be considered superfluous. The incorporation of albumin and blood lactate levels, both of which exhibit substantial predictive capacities, into mortality scoring systems will enhance the predictive capabilities of stated scoring systems.

## Figures and Tables

**Table 1:** Demographic and clinical features of pediatric patients admitted to the PICU who either survived or did not survive.

Patients (*n* = 942)	Survivors (*n* = 868)	Non-Survivor (*n* = 74)	*p*
Gender female, *n* (%)	355 (40.9)	29 (39.2)	0.437
Age, month	32 (9–100)	36 (8–124)	0.618
Sepsis/septic shock, *n* (%)	135 (15.6)	38 (51.4)	**<0.01**
Length of PICU stay, d	4 [2–10]	5 [2–12]	0.259
Length of hospital stay, d	14 [6–30]	6 [2–14]	**<0.01**
Invasive mechanical ventilation, *n* (%)	633 (72.9)	70 (94.6)	**<0.01**
Non-invasive mechanical ventilation, *n* (%)	259 (29.8)	7 (9.5)	**<0.01**
Vasoactive use, *n* (%)	214 (24.7)	70 (94.6)	**<0.01**
Underlying heart disease, *n* (%)	116 (13.4)	15 (20.3)	0.075
Underlying cancer, *n* (%)	92 (10.6)	20 (27)	**<0.01**
Underlying neurologic disease, *n* (%)	216 (24.9)	16 (21.6)	0.32

Values are expressed as mean (%), median [interquartile range], PICU: pediatric intensive care unit.

**Table 2:** Area under the curve (AUC) of receiver-operating characteristics curve (ROC) and respective confidence interval (CI) values of the biological markers and mortality predictors.

Patients (*n* = 942)	Survivors (*n* = 868)	Non-Survivors (*n* = 74)	*p*	AUC	CI (95%)
PRISM-4 DP	1.6 [0.69–4.1]	31.4 [11.41–64.13]	**<0.01**	0.923	0.894–0.952
PIM-3 DP	2.51 [1–6.13]	31.34 [12.75–66.48]	**<0.01**	0.896	0.855–0.937
CRP, mg/L	11 (2–51.2)	82 (13.57–67.96)	**<0.01**	0.728	0.667–0.789
Albumin, g/dL	3.4 (2.97–3.81)	2.73 (2.33–3.13)	**<0.01**	0.795	0.747–0.842
Lactate, mmol/L	1.6 (1.1–2.6)	4.35 (2.7–8.17)	**<0.01**	0.798	0.737–0.860
CRP/Albumin	3.13 (0.56–15)	28.63 (4.88–81.92)	**<0.01**	0.751	0.694–0.808

Values are expressed as median [interquartile range], PRISM-4 DP: Pediatric Risk of Mortality Death Probability, PIM-3 DP: Pediatric Index of Mortality Death Probability, CRP: C-reactive protein.

**Table 3:** Clinical and demographic characteristics of critically ill septic and non-septic children.

Patients (*n* = 942)	Non-Septic (*n* = 769)	Septic (*n* = 173)	*p*
Gender female, *n* (%)	320 (41.6)	64 (37)	0.151
Age, month	28 (9–96)	52 (9.5–127)	**<0.01**
PRISM-4 DP	1.21 [0.6–3.43]	8.2 [3.08–24.2]	**<0.01**
PIM-3 DP	2.26 [0.96–5.95]	7.57 [3.18–21.2]	**<0.01**
CRP, mg/L	8 (1.7–32.1)	103 (29–227.4)	**<0.01**
Albumin, g/dL	3.43 (3.03–3.84)	2.9 (2.45–3.3)	**<0.01**
Lactate, mmol/L	1.65 (1.1–2.6)	2.2 (1.25–4.25)	**<0.01**
CRP/albumin ratio	2.27 (0.48–9.98)	35.39 (8.69–84.7)	**<0.01**
Length of PICU stay, d	3 [2–8]	8 [4–19]	**<0.01**
Length of hospital stay, d	11 [6–24]	22 [11–47]	**<0.01**

Values are expressed as *n* (%), median [interquartile range], PRISM-4 DP: Pediatric Risk of Mortality Death Probability, PIM-3 DP: Pediatric Index of Mortality Death Probability, CRP: C-reactive protein.

**Table 4:** Survival and non-survival characteristics of critically ill pediatric sepsis patients.

Patients (*n* = 173)	Sepsis Survivors (*n* = 135)	Sepsis Non-Survivors (*n* = 38)	*p*
Gender female, *n* (%)	50 (37)	14 (36.8)	0.570
Age, month	54 (12–126)	38 (8–133)	0.739
PRISM-4 DP	5.72 [2.4–14.61]	31.67 [11.38–64.84]	**<0.01**
PIM-3 DP	6.1 [2.68–15.8]	25.05 [8.76–52.1]	**<0.01**
CRP, mg/L	96.5 (25.3–208.3)	193.5 (56.02–277.17)	0.015
Albumin, g/dL	3.05 (2.62–3.44)	2.52 (2.24–2.90)	**<0.01**
Lactate, mmol/L	1.8 (1.2–3.3)	4.35 (2.87–7.20)	**<0.01**
CRP/albumin ratio	28.02 (7.60–74.76)	67.57 (23.29–122.45)	**<0.01**

Values are expressed as *n* (%), median [interquartile range], PRISM-4 DP: Pediatric Risk of Mortality Death Probability, PIM-3 DP: Pediatric Index of Mortality Death Probability, CRP: C-reactive protein.

**Table 5:** Calculating the area under the curve (AUC) and confidence interval (CI) for biological markers and mortality predictors in children with sepsis using receiver operating characteristics (ROC) curves.

Patients (*n* = 173)	Sepsis Survivors (*n* = 135)	Sepsis Non-Survivors (*n* = 38)	*p*	AUC	CI (95%)
PRISM-4 DP	5.72 [2.4–14.61]	31.67 [11.38–64.84]	**<0.01**	0.784	0.700–0.869
PIM-3 DP	6.1 [2.68–15.8]	25.05 [8.76–52.1]	**<0.01**	0.753	0.657–0.849
CRP, mg/L	96.5 (25.3–208.3)	193.5 (56.02–277.17)	0.015	0.629	0.527–0.731
Albumin, g/dL	3.05 (2.62–3.44)	2.52 (2.24–2.90)	**<0.01**	0.734	0.650–0.818
Lactate, mmol/L	1.8 (1.2–3.3)	4.35 (2.87–7.20)	**<0.01**	0.758	0.666–0.849
CRP/Albumin	28.02 (7.60–74.76)	67.57 (23.29–122.45)	**<0.01**	0.659	0.560–0.759

Values are expressed as median [interquartile range], PRISM-4 DP: Pediatric Risk of Mortality Death Probability, PIM-3 DP: Pediatric Index of Mortality Death Probability, CRP: C-reactive protein.

## Data Availability

The data presented in this study are available on request from the corresponding author.
